# Supplementary material for: Are carbon emissions trading and green financial instruments synergistic? -Comprehensive quantitative research based on content analysis
Source: PLoS One. 2024 Mar 7;19(3):e0298601. doi: 10.1371/journal.pone.0298601 (PMC10919601; doi:10.1371/journal.pone.0298601)
Supplement: S1 Data — (ZIP) [file pone.0298601.s001.zip › Data/地级市Z_F工作报告.zip!/║■─╧/╘└╤⌠╩╨/└┤╫╘╣1⁄2╓┌║┼í╛┬φ┐╦╩2╛▌═°í┐_2018.docx]

各位代表：

现在，我代表市人民政府，向大会报告政府工作，请予审议，并请市政协委员和其他列席人员提出意见。

2017年：“四大会战”强力开启岳阳发展新局面

2017年，在中共岳阳市委的坚强领导下，在市人大、市政协的监督支持下，我们全面贯彻党中央、国务院和省委、省政府各项决策部署，认真践行“五大发展理念”，大力实施创新引领开放崛起战略，积极应对“两厂”周期性停产检修，全力迎战严重洪涝灾害，主动接受中央环保督察、国务院第四次大督查、国务院安委会综合督查、统计执法检查、脱贫攻坚实地考核等一系列高规格督查考核，坚持以“四大会战”统揽新增长极建设，预计全市GDP、固定资产投资、规模工业增加值、社会消费品零售总额、居民人均可支配收入分别增长6.7%、13.5%、5.5%、10%和9.5%，经济社会发展处于稳健区间。

产业发展迈出坚实步伐。始终保持抓产业发展的坚定信心和战略定力，加快构建多点支撑、多元发力的产业发展新格局，为新增长极建设提供了强劲动能。产业园区走势强劲。强力推进园区建设大会战，经开区、绿色化工产业园有望晋升千亿产业园区，将实现我市千亿产业园区“零”的突破；全市园区规模工业增加值占比71.3%，同比提高4.3个百分点。继续加大标准化厂房建设力度，新建厂房260万平方米以上，民间资本投资超过80%，入驻企业125家，企业入驻率达到82%，标准化厂房成为吸引企业入驻的“强大磁场”。对园区产业招商实行“一票否决”，没有完成5亿元、10亿元工业项目招商任务的，其县市区党政主要领导和园区主要负责人不评先、不提拔、不重用，形成了产业招商倒逼机制。部署开展“产业招商月”活动，6—7月集中时间、集中精力专题招商，全市10个产业园区累计签约产业项目325个，合同引资787亿元，其中30个5亿元以上的工业项目开工18个，其他12个近期可以落地，特别是和润粮油、哈工大机器人、东方雨虹二期、经卓新材料、数博环球、九鼎产业园、科伦研究院三期、10万吨丁二烯、泰格林纸改扩建等一批投资过10亿元的大项目、好项目成批入驻，是近年来引进产业项目数量最多、规模最大、质量最高、成效最好的一年。商贸物流加速扩张。加大商贸物流项目引进和建设力度，步步高·新天地、南翔万商国际商贸物流城一期、金悦洋商业公园一期等一批规模大、品位高的项目建成开业；友阿城市商业综合体、红星美凯龙、海吉星农产品物流园、临港国际汽车城、白杨湖总部经济港、空港产业园等一批潜力大、前景好的项目扎实推进，岳阳商贸物流进入新一轮提质扩张期，为打造千亿物流产业提供了坚实支撑。全域旅游持续升温。加快建设全域旅游目的地城市，洋沙湖国际旅游度假区、屈子文化园顺利开园，洞庭湖博览园、天岳幕阜山等文旅项目强力推进，东方铂尔曼大酒店入驻岳阳，成功举办湖南国际旅游节等节会活动，旅游人气持续高涨，全年接待旅游总人数5200万人次，平江获批国家级旅游业改革创新先行区。现代农业稳步发展。环洞庭湖基本农田建设重大工程全面完成，累计建成高标准农田5.66万公顷，新增耕地6791公顷，洞庭湖“天下粮仓”地位进一步巩固。全面完成第三次全国农业普查和土地确权登记，流转土地210.5万亩。规模以上农产品加工企业发展到493家，新增“三品一标”农产品62个。屈原成功创建第一批国家农业可持续发展试验示范区，君山获评首批国家农产品质量安全区，平江获评全国电子商务进农村综合示范县，岳阳县获评全国农业农村信息化示范基地、全国第一批畜牧业绿色发展示范县，湘阴、华容获评“中国好粮油”行动示范县。

开放崛起站上新的起点。坚持“敞开门户促发展”，强力推进港口建设大会战，岳阳开放发展按下了“快进键”，跑出了“加速度”。开放平台越来越多。在推动“一区一港四口岸”高效运营基础上，岳阳成功纳入中国（湖南）自由贸易试验区申报范围；成功设立湘欧快线国际货运班列站点；成功进入全国多式联运示范项目“笼子”；成功建成投运岳阳（汨罗）公路口岸，开放平台优势进一步凸显。开放力度越来越大。全面兑现开放型经济发展“1+2”政策组合文件，加大外贸企业奖补力度，88家外贸企业“破零倍增”。全年进出口总额增长70 %，跃居全省第3位；集装箱吞吐量突破40万标箱，首次超过九江、宜昌。岳阳成功入围“中国外贸百强城市”榜单，居全省各市州第1位。开放范围越来越广。与法国大圣富瓦市缔结为友好城市；首次在俄罗斯、蒙古举办产业对接洽谈会，境外投资企业增至36家，覆盖20多个国家；引进股份制银行3家，设立农商行2家，新增上市企业1家。岳阳开放发展态势更加稳健、动能更加强劲。

基础设施建设实现超常突破。加快完善基础设施体系，坚定不移推进“双百工程”和土地依法征收，113个重大建设项目完成投资683.6亿元，同比增长39%，102个重大前期项目开工26个。便捷高效的交通体系正在加快形成。启动近年来实施项目最多、建设里程最长、投资额度最大的交通建设大会战，新增一级公路118公里，总里程跃居全省第2位，城区“30分钟交通圈”、市域“1小时交通圈”初具雏形。杭瑞高速洞庭湖大桥、皇姑塘立交桥、临湖公路、湘北大道、空港大道等12条主干道路建成通车；三荷机场、蒙华铁路等一批重大项目扎实推进。特别是赶山东路、G240岳阳县城至湘阴公路，从开工到通车只用了不到一年时间，刷新了“岳阳速度”，其中G240岳阳县城至湘阴公路全长64公里，是我市一次性建成里程最长的一级公路，也是湖南“十三五”第一条实施、第一条完工的干线公路。保障有力的水利体系正在全面构建。坚持“确有需要、生态安全、可以持续”的原则，加速加力推进农田水利建设，重点垸堤防加固、小型农田水利重点县建设、渠道清淤整治、三峡后续一期河道整治、城市防洪应急处险等一批水利项目扎实推进，修复水毁工程4700处，较好解决了水利防汛保安、农田灌溉“最后一公里”的问题。多元并举的能源体系正在逐步健全。平江抽水蓄能电站开工建设、华电平江电厂、神华国华岳阳电厂进展顺利，投产输变电项目12个，并网投运20兆瓦以上光伏发电站10个，增效扩容改造农村水电项目20个。同时，启动“宽带中国·智慧岳阳”建设，完成1181个行政村宽带网络升级改造。通过织密织牢交通、水利、能源、信息“四张网”，基础设施承载能力明显增强。

改革创新取得重大进展。更加注重向改革要动力、向创新要活力，结合岳阳实际在重点领域和关键环节革故鼎新、推陈出新，着力破解了一批发展瓶颈。坚定不移推进改革试点。以供给侧结构性改革为主线，纵深推进9类145项重点改革事项。商事制度“放管服”综合配套改革扎实推进，新增市场主体4.3万户；承担行政职能事业单位改革、公共资源进场交易等走在全省前列；屈原农村改革集中区创建取得新的成效；岳阳被确定为全省唯一深化供销合作社综合改革示范市。这些改革和试点，有效破解了岳阳发展的机制障碍，降低了岳阳发展的制度成本。坚定不移实施创新引领。坚持通过解放思想来解决问题，通过推动创新来推进发展。加强科技创新，继续实施“巴陵人才工程”，启动实施“百名专家投智岳阳”行动，新增省级院士工作站、省级科技企业孵化器和重点实验室17家，新增高新技术企业68家，高新技术产业增加值占比提高到20%。加强制度创新，研究出台“园区十条”“非公十条”“开放十条”“金融十条”和“实体经济二十条”，这是岳阳在深化供给侧结构性改革上推出的具体举措，是在实施创新引领战略上打出的一套政策“组合拳”，有力撬动了实体经济发展提速提效。

生态治理保持高压态势。坚持绿色发展理念不动摇，以壮士断腕、背水一战的决心和勇气，狠抓生态环境整治，查处环境违法案件622起，形成了环境整治严控严管的高压态势、常态长效的工作机制。强力推进大江大湖综合治理。统筹抓好湘江保护和治理省“一号重点工程”、洞庭湖水环境综合治理“五大专项行动”，攻克了一批生态环境“老大难”问题。率先全省开展公职人员涉砂经营专项整治，集中清查重点对象716名，均按规定分类处置到位，这项工作走在全省前列，得到省委、省政府高度肯定。启动涉矿等6类经营性活动专项整治，清查参与涉矿等经营活动公职人员432名，年内全部清退到位。组织开展“洞庭湖生态环境突出问题集中整治月”行动，国庆期间，相关领导和有关单位不放假、不休息，集中精力抓整改，牢牢把握了整治工作主动权。启动东风湖新区治理与开发，马壕污水处理厂二期、沿湖截污干管、湖东路、上湖景观带等快速推进，城市内湖水环境持续改善。全面推行“河长制”，市县乡村四级河长制实现全覆盖。强力推进中央环保督察问题整改。主动适应环保新要求，从严从实整改中央环保督察反馈的突出问题，中心城区完成黑臭水体整治20处，关停砂石堆场44处，取缔粘土砖窑87家，退养洞庭湖区养殖场2513户79.8万平方米，君山公园后湖和采桑湖生态修复、黄盖湖网箱养殖取缔、环湖生活垃圾堆放点清理等16项中央环保督察年度整改任务完成销号，华容河水环境整治、工业园区污水处理设施建设等有序推进。同时，统筹推进“海绵城市”试点、城市污水管网建设和“乔木进城”三年行动，岳阳成功入选首批全国气候适应性城市建设试点。岳阳大江大湖的特色，既是生态资源优势，更是生态环保压力，在中央环保督察这场“大考”中，我们思想认识前所未有，付出努力前所未有，工作成效前所未有，全市上下以“保护青山绿水”的历史责任和“建功必须有我”的使命担当，吹响了保护生态环境的“集结号”，打响了环保问题整改的“歼灭战”，美丽岳阳的底色更加鲜明、更加靓丽！

人民生活得到明显改善。坚持促发展与惠民生并重，加快释放民生红利，20项27件省市实事全面完成，城乡居民人均可支配收入分别达30025元和14234元，全面小康实现程度提高到96%，人民群众幸福感、获得感进一步增强。脱贫攻坚方面：强力推进脱贫攻坚大会战，坚持以“一超过两不愁三保障”为重点，实施易地扶贫搬迁项目29个，7002户贫困户喜迁新居，1.2万贫困户享受危房改造政策；全市贫困村种养产业、光伏产业村级覆盖率分别达92.4%和98.8%；新增扶贫小额信贷6.2亿元；贫困人口实现就业近2万人；纳入社保兜底保障对象1.8万人；发放教育助学资金1.4亿元；“三个一批”健康扶贫政策全面落实。全市6.6万人摆脱贫困，187个村脱贫出列，贫困发生率由4.29%下降至2.29%，交出了一张优异的“成绩单”。防汛救灾方面：全市上下众志成城、严防死守，夺取了防汛救灾的全面胜利，奋力打赢了全省汛情最严峻、最复杂、最危急的湘阴沙田垸保卫战，经受住了严重洪涝灾害的历史考验，得到了省委、省政府的高度肯定；在灾害特别严重的平江、汨罗、临湘、岳阳县等地，积极开展救灾补损和灾后重建，确保了大灾之年群众生产生活没有大震荡。“两改”工作方面：中心城区“十大片区”征拆基本清零，2017年新启动55个棚改项目，超额完成省定目标，5000套限价商品房全面开工，2万多户市民群众摆脱“蜗居”历史；300个农村规划集中建房点开工203个，建成103个，竣工6875户，惠及3.5万农民群众，农村“有新房无新村”的现状正在加快改变。社会事业方面：教育事业繁荣发展，中心城区中小学校“六建六扩”扎实推进，市十六中、市十八中、北港小学等建成投入使用，岳阳雅礼、湘一南湖等优质民办学校布局岳阳，全年新建中小学校51所、农村公办幼儿园17所，改薄学校785所，广大群众反映强烈的“大班额”问题正在有效化解。岳阳选手夺得全运会摔跤冠军，实现了我市全运会金牌“零”的突破。文化惠民工程扎实推进，市图书馆新馆主体工程完工，村级综合文化服务中心覆盖率达84%，群众性文体活动更加丰富多彩。“健康岳阳”建设深入推进，加快医疗资源布局调整，市妇女儿童医院、市中医院基本医疗大楼、市二医院全科医生培养大楼等加快建设，建成医联体4个，2.6万残疾人享受精准康复服务。农村地区殡葬改革持续深化。社会保障提标扩面，城乡低保标准人均每年分别提高到5280元和3120元，企业退休职工养老金人均每月提高到2061元；新增城镇就业5.4万人。社会治理方面：安全生产、综治维稳、信访工作进一步加强，社会大局和谐稳定，十九大安保维稳工作被省公安厅通令嘉奖。出台史上最严禁炮令，社会反响良好。禁拆治违工作经验、“12345+110+网格化”社会治理新模式在全国推介，岳阳成功蝉联全国文明城市，特别是在历经8年创建之后，2017年岳阳成功摘得“全国社会治安综合治理优秀市”的金字招牌。总体来看，这一年是岳阳近年来棚改力度最大的一年，是建成中小学校最多的一年，是医疗资源扩张最快的一年，岳阳人民享有的民生“礼包”越来越多、越来越大！

民主法制建设取得长足进步。主动接受市人大法律监督、工作监督和市政协民主监督，配合市人大常委会出台《岳阳历史文化名城保护条例》《岳阳市城市规划区山体水体保护条例》，开启了岳阳地方立法的新篇章；承办人大代表建议和政协提案497件，办结率、满意率分别达100%和99.8%。坚持依法行政，修订完善“岳阳市政府工作规则”，严格落实“两清单两目录”，系统制定政府债务防控、融资平台管理、土地挂牌出让、工程项目监管等规范性文件，不断扎紧行政权力运行的制度“篱笆”，做到了既让权力“进笼子”，又让权力“晒太阳”。大力简政放权，推行“两集中两到位”，行政审批事项减少到170项，精简55.6%；推进“互联网+政务服务”，市政府政务服务中心获“全国优秀政务大厅精细管理创新奖”，总分居全国第3位，成为全省唯一获此殊荣的市州；12345热线全面扩容，38条非紧急类政府服务热线成功整合为“一号通”。顺利完成村（居）“两委”换届选举，全面完成农村综合服务平台建设。加强党风廉政建设和反腐败斗争，推行“互联网+监督”，扩大了公众参与面；开展“吃空饷”专项治理，取得了阶段性成效。推进“两学一做”学习教育常态化、制度化，大力弘扬岳阳“四种精神”，坚定不移地反“四风”、正行风，积极开展“刹人情歪风、治婚丧陋习、树文明新风”专项行动，推行“婚事新办、丧事简办、其他事项不办”，倡导形成勤俭节约、文明健康的社会新风尚，中央电视台《新闻联播》栏目向全国予以推介。

其他各项事业繁荣发展，国防动员、武警、消防、人防等工作实现了新进步；统计、史志、档案、国安、保密、老干老龄、民族宗教、科协、气象、联络接待、公积金、移民、烟草、石油、盐业、邮政、通信等工作取得了新成绩；工会、共青团、妇联、侨联、工商联、台办、贸促及各民主党派、无党派人士等工作作出了新贡献。

过去一年，岳阳面临的挑战比预料的大，但收获的成果比预想的多，办成了一些大事，解决了不少难题，有的是多年想办而没有办成、多年想解决而没有解决的，岳阳加快发展谋篇布局的架势已经拉开，赶超跨越的态势正在形成，成绩来之不易，人民有目共睹。这些成绩的取得，是省委、省政府和市委正确领导的结果，是市人大、市政协监督支持的结果，是全市人民团结奋斗的结果。特别是广大干部群众，大力弘扬不怕困难、不畏艰辛、不甘人后的拼搏精神，广大公职人员始终保持冲锋陷阵、一线作战、大干快上的工作状态，以爬坡过坎、攻坚克难、负重奋进的昂扬锐气，顶住压力加油干，励精图治抓发展，一年来大家工作都非常辛苦，付出了很多努力，流下了很多汗水，作出了很多牺牲，这一点市委、市政府是高度认可的，也是充分肯定的！实践证明：我们的队伍是一支顾全大局、维护整体的队伍，是一支勇于担当、乐于奉献的队伍，是一支敢打硬仗、能打胜仗的队伍！只要我们全市一盘棋、上下一条心、拧成一股绳，任何困难都压不垮岳阳人民的脊梁，任何挫折都摧不毁岳阳人民的意志，任何险阻都挡不住岳阳人民前进的脚步！在此，我代表市人民政府，向全市人民，向所有参与和支持岳阳改革发展的社会各界人士，表示衷心的感谢和崇高的敬意！

过去一年，尽管我们付出了很多努力，但由于“两厂”停产检修和遭受严重洪涝灾害，加之我们主动适应环保新要求，加快淘汰落后产能，坚决关停清理了204家不达标规模企业，部分经济指标没有达到年初目标，但如果剔除这些因素的影响，我市年初确定的目标是可以实现的。一方面，这说明了我们对2017年发展形势估计还不充分，把握还不精准；另一方面，这也是岳阳发展过程中一种特殊性、阶段性、暂时性现象，只要我们正确应对，岳阳经济社会发展长期向好的基本面是不会改变的。

在充分肯定过来工作成绩的同时，我们也要清醒地看到存在的问题和不足。当前，岳阳发展周期性矛盾和结构性矛盾相互叠加，短期性问题和长期性问题彼此交织，经济形势仍然复杂，工作压力仍然较大：一是新增长极建设后劲还不够强，重大产业项目、优质民营企业、开放型经济主体总量仍然偏少；二是经济质量和效益还不够高，产业结构不优、地方财力不强、县域经济不强等短板仍然存在；三是城乡发展还不够平衡，基础设施建设、社会保障体系、公共服务体系差距仍然较大；四是各类风险隐患还比较多，生态治理、安全生产、信访维稳和政府债务、金融风险化解等压力仍然存在；五是干部作风建设还有待加强，少数干部法纪红线意识、廉政底线意识仍需进一步提高，反“四风”、转作风仍需进一步从严从实等等。对这些问题，我们一定高度重视，认真加以解决。

2018年：向着新增长极建设宏伟目标坚实迈进

2018年，既是我市贯彻落实党的十九大精神的开局之年，也是决胜全面建成小康社会的关键之年，更是完成脱贫攻坚任务的决战之年。中国特色社会主义进入新时代，必然赋予新使命、开启新征程；岳阳新增长极建设站在新起点，必须顺应新形势、把握新要求。全市上下要牢固树立新的发展理念，切实转变工作思路，坚定不移推进湖南发展新增长极建设，确保岳阳在建设富饶美丽幸福新湖南的宏伟征程中走在前列、多作贡献。我们要更加注重质量效益，坚决淡化速度情结。当前，我国经济已由高速增长阶段转向高质量发展阶段，推动高质量发展是保持经济持续健康发展的必然要求。各级各部门必须把提高经济质量和效益摆在更加突出的位置，坚持扩总量与优存量并重，保持稳增长与调结构平衡，不断巩固“稳”的基础，持续增强“进”的动能，全力推动高质量发展，努力实现调速不减势、量增质更优。我们要更加注重创新引领，坚决打破惯性思维。中央持续加大“去杠杆”力度，信贷投放渠道收窄，“拼投入”“铺摊子”“摊大饼”的传统增长模式已经难以为继。各级各部门要坚定不移实施创新引领战略，把人才作为创新的第一资源，统筹推进以科技创新为核心的全面创新，努力推动经济增长由投资驱动向创新驱动转变、从外延式增长向内涵式发展转变。我们要更加注重生态优先，坚决克服路径依赖。岳阳资源环境约束日益趋紧，生态保护压力日益加大，传统的粗放型增长路子只会越走越窄。各级各部门要正确处理好经济发展与环境保护的关系，切实强化“绿水青山就是金山银山”的理念，坚决不上“高污染、高消耗”的项目，坚决不走“先污染、后治理”的老路，为岳阳留住青山绿水、留下蓝天白云。我们要更加注重为民利民，坚决防止政绩偏差。人民对美好生活的向往，就是我们的奋斗目标。各级各部门要牢固树立“以人民为中心”的发展理念，把民情民意作为政府工作的“风向标”，坚持从群众呼声最强烈的事情抓起、愿望最迫切的事情办起，努力把更多的财政增量转化为老百姓实实在在的生活质量，真正让岳阳成为闻者向往、来者依恋、居者自豪的民生福地！

2018年政府工作总体要求是：坚持以习近平新时代中国特色社会主义思想为指导，全面贯彻落实党的十九大精神和中央、省委、市委经济工作会议精神，坚持稳中求进的工作总基调，紧扣社会主要矛盾变化，按照高质量发展的要求，统筹做好稳增长、促改革、调结构、惠民生、防风险各项工作，坚决打好防范化解重大风险、精准脱贫、污染防治攻坚战，深入实施创新引领开放崛起战略，强力推进“四大会战”，加快建设国家区域性中心城市，加快全面建成小康社会，在新增长极建设宏伟征程中迈出更加坚实的步伐。

2018年全市经济社会发展目标是：地区生产总值增长8%左右；固定资产投资增长13%；规模工业增加值增长7.5%；社会消费品零售总额增长11.5%；一般公共预算收入增长9%；居民人均可支配收入增长9%以上，居民消费价格指数涨幅控制在2%以内。

主要任务是：

一、坚持经济提质提效，打造岳阳发展的“升级版”

建设现代化经济体系是岳阳经济发展的时代主题。我们要按照省委、省政府的部署，积极开展“产业项目建设年”行动，把“质量第一、效益优先”摆在更加突出的位置，着力增强岳阳经济质量优势。

落实“园区十条”，做强工业经济。工业强则园区强、园区强经济强。岳阳要稳固湖南第二经济强市的地位，必须依靠大工业、大园区作支撑。我们要进一步树牢“工业强市、园区兴工、项目立园”的发展理念，强力推进园区建设大会战，严格落实“园区十条”，以工业经济的先行突破带动市域经济的整体提升，在现有2个千亿园区的基础上，力争到2020年，技工贸总收入过1000亿元的园区再增加1个以上、500—1000亿元的园区达到3个以上。要坚持项目立园，持续推进产业招商。经开区、城陵矶新港区、绿色化工产业园3个重点园区，每年至少要引进一个10亿元以上的工业项目；其他7个省级以上园区，每年至少要引进一个5亿元以上的工业项目，全市全年要引进亿元以上产业项目100个以上，没有完成产业招商任务的严格实行“一票否决”。我们要牢牢抓住战略性产业项目不放手，实行市级领导“一对一”盯项目制度，力争三鼎集团己内酰胺、中国邮航速递物流、岳阳市电子产业园等10个战略性项目尽快落地开工；同时，加快推进350万吨化工型炼油等重大项目，以大项目支撑园区大发展。要坚持园区兴工，持续提高园区质量。坚定不移走“特色化”“差异化”的产业园区发展路子，鼓励引导各园区结合自身资源禀赋和实际情况，重点发展2—3个特色鲜明、走势强劲、集群发展的主导产业，加快引进一批关联度高、配套性强的上下游企业，进一步提高园区优势产业集聚度，推动产业园区由“小而散”向“大而强”转变。要坚持工业强市，持续推动产业升级。做大做强优势产业，依托“两厂”产品和技术优势，加快东方雨虹二期、10万吨丁二烯、5万吨SEBS等项目建设，推动石化产业优势优先发展。培育发展新兴产业，突出抓好哈工大机器人、经卓新材料、数博环球、科伦研究院三期、中樱汽车等项目建设，推动装备制造、生物医药、军民融合、节能环保等新兴产业加快发展，力争新兴产业增加值占比提高2个百分点以上。升级改造传统产业，加快和润粮油、九鼎产业园、泰格林纸改扩建、中储粮油脂基地、石膏建材产业园等项目建设，进一步提高食品、饲料、建材等产品的附加值。要通过几年持续不断的努力，以点对点的招商、硬碰硬的考核、实打实的项目，推动工业转型升级，推动园区提质扩张，推动岳阳由工业大市向工业强市坚实迈进。

落实“开放十条”，做大港口经济。开放是岳阳发展的潜力所在、希望所在，岳阳开放的大门只能越打越开，开放的步子只能越迈越大。我们要主动融入“一带一路”和长江经济带建设，继续推进港口建设大会战，严格执行“开放十条”，加快建设全国现代物流创新发展城市、长江中游交通物流枢纽城市，确保岳阳在全省开放崛起战略中“打头阵”“当先锋”。突出抓好城陵矶新港二期和综保区二期建设，推进长江、湘江航道疏浚和口岸外贸联检锚地建设，建成运营岳阳三荷机场，加快空港产业园建设，尽快形成“双港驱动”发展的新格局。突出抓好中国（湖南）自由贸易试验区申报跟进工作，尽快将岳阳申报片区建成“准自贸区”，加快推进白杨湖总部经济港、湘欧快线国际货运班列站点、跨境电商等开放平台建设，积极申报进境水果指定口岸，全面提升岳阳开放平台优势；继续深化与上港集团合作，引导进出境货物从城陵矶口岸进出，力争集装箱吞吐量达到45万标箱。突出抓好长江沿线、湘江内河等港口岸线资源整合，优化拓展国际直达航线，加快推进多式联运示范项目建设。深入落实“千亿物流产业行动方案”，做大做强海吉星农产品物流园、中粮物流产业园、临港国际汽车城等物流园区，着力引进一批外向型企业沿江沿港布局，培育壮大一批5A级物流企业，尽快把物流业打造成为我市第三个千亿产业。

落实“非公十条”，做优民营经济。深入开展企业降成本行动，坚决兑现“非公十条”，以政策“杠杆”撬动民营经济加速扩张。继续实施30家重点民营企业帮扶行动，下定决心帮助民营企业减负减荷、减压减困，推动民营企业轻装上阵、轻松前行。组织实施“岳商回归”工程，继续开展“引老乡、回故乡、建家乡”活动，大力引进一批寓外乡友投资岳阳。积极稳妥处置“僵尸企业”，加快盘活存量用地，最大限度引导资源要素向成长性好、带动性强的优质企业配置。鼓励支持民营资本加快进入现代服务业领域，大力培育工业设计、电商物流、服务外包等生产性服务业；加快发展全域旅游、文化创意、娱乐休闲、健康养老等生活性服务业，为民营企业发展拓展更多的空间、开辟更大的天地。我们要努力营造企业家健康成长的环境，大力弘扬优秀企业家精神，更好发挥企业家作用，坚决拆除市场准入的“门槛石”，坚决破除政务服务的“玻璃门”，坚决扫除行政审批的“中梗阻”，坚决清除项目建设的“拦路虎”，切实解除企业家的“后顾之忧”，稳定企业家投资和经营预期，让企业家在岳阳放心投资、舒心创业、安心发展。我们也希望广大客商更多关注岳阳、了解岳阳、投资岳阳，在岳阳觅得更多商机、成就更大梦想！

落实“金融十条”，做实实体经济。经济发展的根基在实体、在产业。要严格落实市委、市政府即将出台的《关于加快实体经济发展的若干意见》，把发展经济的着力点放到实体经济上来。以“金融十条”为抓手，精准对接“四大会战”和9大优势产业，推动金融回归服务实体经济的本源，把更多金融资源配置到经济社会发展的重点领域和薄弱环节。积极发展现代金融，加快资产资本化、资本证券化，推进企业上市和股权挂牌，提高直接融资比重。大力培育各类金融机构，引进1—2家股份制商业银行。拓宽保险服务领域，积极发展巨灾保险、农业保险、责任保险和商业养老保险，加快构建多层次、广覆盖的民生保险体系。

二、坚持项目为重为要，牵住岳阳发展的“牛鼻子”

项目建设是经济发展的重要抓手。我们抓发展首先必须抓项目，要在抓好重大产业项目建设的同时，加快推进基础设施项目建设，以大项目带动大投资、推动大发展，以一个一个的项目筑起岳阳新增长极建设的“万丈高楼”。

狠抓交通项目建设。深入推进交通建设大会战，进一步完善综合立体交通运输体系。扎实做好常岳九铁路、松杨湖铁路专用线等前期工作；配合抓好岳望高速建设，加快推进平益高速、城陵矶高速、杭瑞高速金凤桥连接线建设；升级改造S308南江至龙湾段公路、S210汨杨公路，建成运营G536汨罗段改线、长康南路等干线公路，力争新增二级以上干线公路100公里；运用市场手段，引进民营资本参与G107改线提质工程，全方位打造外部配套成环、内部纵横成网的大交通格局。

狠抓水利项目建设。全面推进“9936”工程，着力补齐水利设施“短板”。提高水利防汛抗灾保障能力，突出抓好重点垸堤防加固，完成83座小型病险水库除险加固，着力夯实水利防汛保安基础；提高水利生态涵养保障能力，加快推进黄盖湖综合治理、梅溪港河治理、华洪运河长江补水工程、黄梅港河修复、南湖电排站扩容改造等项目建设，切实改善河湖水体环境；提高水利农田灌溉保障能力，实施洞庭湖区沟渠塘坝清淤增蓄专项行动，清淤大中型渠道622条1417公里，确保农田灌溉“水到渠成”；提高水利安全饮水保障能力，继续实施农村饮水安全巩固提升工程，进一步提高农村集中供水率、自来水普及率、水质达标率，为岳阳人民生产生活构建一道放得心、靠得住的水利“保障网”。

狠抓能源项目建设。要加快推进平江抽水蓄能电站、华电平江电厂、神华国华岳阳电厂等重大能源项目建设；全面推进农网升级改造，加快7个重点输变电工程建设，新增水电装机容量3600千瓦；大力推进光伏发电和风能、生物质能利用，进一步提高能源供应保障能力，加快建设现代能源体系。同时，统筹推进信息基础设施建设，推动岳阳向“5G时代”“互联网+”时代加速迈进。

三、坚持城乡互促互融，构建岳阳发展的“新格局”

统筹城乡发展是解决发展不平衡不充分问题的有效抓手。我们要坚定不移走城乡融合发展的路子，坚定不移实施乡村振兴战略，加速加力推进城乡一体化。

加快构建大岳阳城市圈。当前，岳阳正处在城镇化中期阶段，城市发展仍处在上升通道，我们要认真抓好顶层设计，在科学划定城市开发边界控制线的同时，按照“框定总量、限定容量、盘活存量、做优增量、提高质量”的要求，着力提高“大岳阳城市圈”在全市经济社会发展中的首位度，加快建设国家区域性中心城市。稳步拓展城市空间。围绕打造200万人大城市圈目标，加快构建“1+4+2”城市组团。全方位拉开城市“骨架”，强力推进北环线、西环线、联港路、求索西路延伸段建设，确保建成奇康路、汴河园路等一批交通要道，尽快形成中心城区30分钟交通圈。加快提升城市品位。加大城市“双修”力度，科学把握城市棚改规模、力度和节奏，统筹抓好棚改片区规划、招商与建设，全面推进杨树塘、四化建、天灯咀、刘山庙和洞庭新城、高铁新城等重点棚改片区建设，力争把一个一个的城市“棚户区”打造成为城市“精品区”。突出抓好东风湖新区开发与建设，坚持“治理与开发相结合”，按照“生态、高端、现代、时尚”的定位和“生态优先、产城融合”的要求，尽快将其打造成为岳阳城建的新标杆、岳阳城市的新名片。同时，切实抓好“海绵城市”试点，加快推进地下综合管廊建设，继续实施“城市污水管网建设三年行动方案”，统筹城市地上与地下建设，使城市既有“面子”，又有“里子”。严格执行城市规划。发挥规划的战略引领和刚性管控作用，切实抓好“岳阳市城市总体规划”修编，城市规划与建设要坚持“以人为本、尊重自然、传承历史、绿色低碳”，坚决做到不推山、不填湖、不毁绿，不破坏城市年轮、不损害城市文物、不改变城市风貌，最大限度彰显岳阳城市的文化元素、生态要素与和谐因素，让岳阳这座古老而又现代的城市，真正成为岳阳人民的骄傲与自豪！

推进县城城镇扩容提质。要坚持新城扩容与旧城提质、硬件建设与软件建设“两手抓”，每个县城既要适度拓展新城区，又要提质改造老街区，还要统筹抓好城区道路、星级酒店、休闲广场、教育医疗等服务设施，以及绿化、亮化、美化、净化等配套设施建设，全面提高县城品质和服务承载能力。坚持把城镇作为统筹城乡发展的重要节点来抓，在抓好16个国家重点镇建设与发展的同时，每个县还要重点抓好2—3个小城镇建设，着力打造一批旅游型、边贸型、加工型等特色小镇，让更多的特色镇穿点成线、串联成珠，整体提升岳阳城镇的品质与品位。

大力实施乡村振兴战略。全面建成小康社会最大的短板在农村。任何时候我们都要坚持农业农村优先发展，把实施乡村振兴战略作为新时代“三农”工作的总抓手，按照“产业兴旺、生态宜居、乡风文明、治理有效、生活富裕”的总要求，推动乡村振兴战略落地见效。加快发展现代农业。坚持以农民增收为核心，推动农业从增产导向向提质导向转变，突出抓好屈原和华容国家现代农业示范区、洞庭湖绿色食品产业园、台湾农民创业园等农业园区建设，培育发展岳阳黄茶、平江豆干、华容芥菜、汨罗粽子、洞庭小龙虾等地域特色产业。坚持以土地流转为抓手，完善承包地“三权”分置制度，鼓励工商资本下乡村，引导企业主体进农村，以规模化、集约化、企业化方式参与农业生产和农村发展；加快培育一批新型农业经营主体，大力发展多种形式的适度规模经营，积极推进田园综合体建设，促进农村一二三产业深度融合发展。加快建设美丽乡村。目前，我市农村大量存在空心房、危旧房和杂房，既影响农村形象，又占用土地资源，还影响生态环境。2018年，要在全市部署开展农村“空心房”集中整治行动，通过盘活农村土地资源，推动规范村民建房、农村集中建房由点到面、整域推进，新建成30栋以上集中建房点100个以上；突出抓好农村公路提质改造、危桥改造和安防工程建设，加快改造通组公路；突出抓好农村环境治理，以实施农村“厕所革命”为重点，深入推进乡村“清洁工程”。要通过几年坚持不懈的努力，力争岳阳美丽乡村建设进入全省、全国先进行列。加快提升乡村文明。继续开展“刹人情歪风、治婚丧陋习、树文明新风”专项行动，加快农家书屋、农民健身工程等阵地建设，丰富群众精神文化生活。健全农业社会化服务体系和乡村治理体系，努力培养一支懂农业、爱农村、爱农民的“三农”工作队伍。要通过不懈的努力，真正让农业更有奔头、农村更有吸引力、农民更有尊荣感！

四、坚持改革落地落细，增强岳阳发展的“驱动力”

改革是破解发展难题的“金钥匙”。今年是改革开放40周年，改革已进入攻坚期，我们要敢涉“深水区”，敢啃“硬骨头”，加快推动质量变革、效率变革、动力变革，坚决将改革进行到底。

全面深化改革。坚持以供给侧结构性改革为主线，突出提高供给体系质量这个主攻方向，统筹抓好各项改革任务落地。深化经济体制改革。以完善产权制度和要素市场化配置为重点，大力培育新动能，大力降低实体经济成本和制度性交易成本。稳步推进商事制度改革，完成“放管服”改革试点任务；加强财税体制改革，实施绩效管理，完善现代财政制度；实施市场准入负面清单管理模式，继续精简负面清单；加快建立现代企业制度，切实抓好国企“三供一业”和社会职能分离移交工作；推进统计管理体制改革，认真组织开展第四次全国经济普查。深化行政体制改革。继续大力简政放权，加快转变政府职能；推进事业单位改革，促进政事分开、事企分开、管办分离。深化农村综合改革。突出抓好农村土地制度、集体产权制度改革，加大屈原农村改革集中区创建力度，加快君山垦区改革发展，确保成功创建深化供销合作社综合改革示范市。

实施创新引领。坚持创造性转化、创新性发展，把创新摆在发展全局的核心位置、作为引领发展的第一动力，加快形成以创新为引领和支撑的经济体系和发展方式。强化科技创新，加快创建临港国家高新区，新建一批国家级和省级工程技术研究中心、院士工作站等创新平台，新增高新技术企业50家。继续实施“巴陵人才工程”和“四海揽才”计划。大力推进创新创业，鼓励更多的社会主体投身创新创业，在巴陵大地掀起万众创新、大众创业的新热潮。

五、坚持生态严管严治，守住岳阳发展的“生命线”

建设生态文明是永续发展的千年大计。我们要坚定不移走生产发展、生活富裕、生态良好的文明发展道路，统筹山水林田湖草系统治理，推动生态环境总体改善，促进人与自然和谐共处。

加强水体污染防治。坚决落实国家“水十条”，实施最严格的水资源管理制度，全面执行“河长巡河工作方案”，严格实行“一河一策”，强化各级河长目标管理、巡河督查和责任落实，倒逼河长制落地见效。扎实抓好湘江保护和治理省“一号重点工程”、洞庭湖生态环境专项整治“三年行动计划”，加快城区、重点乡镇、工业园区污水处理设施建设，巩固提升大江大湖整治成果。大力推进“一河三湖”及南湖、王家河、汨罗江、新墙河等重点河湖综合整治，加强铁山水库、金凤水库等重点饮用水源地保护，加快完成黑臭水体治理任务，全面改善流域生态环境，还河湖一片安澜，还群众一泓净水。

加强土壤污染防治。坚决落实国家“土十条”，积极实施土壤环境保护工程。突出抓好洞庭湖湿地生态保护和修复，探索推行湿地生态效益补偿试点、湘江流域退耕还林还湿试点，提高湿地生态涵养功能。突出抓好原桃林铅锌矿及周边地区、岳阳县新开钒矿区等重金属污染防治，加强重化工企业、固废填埋场、废弃物堆场等重点部位监管，加快静脉产业园建设，着力恢复土壤结构和功能。突出抓好农业面源污染治理，实施化肥农药“零增长”行动，严格农业生产全过程环境监管，坚决管好岳阳每一寸土地。

加强大气污染防治。坚决落实国家“大气十条”，持续实施大气污染防治行动，全力打赢蓝天保卫战。深入开展加油站油气回收、砖瓦行业污染、餐饮行业油烟污染三大专项整治行动，严格实施燃煤锅炉清洁能源改造，加快淘汰燃煤小锅炉；全面实行城区禁炮，狠抓建筑、道路、交通扬尘污染治理；大力推广新能源汽车；启动大规模国土绿化行动，继续推进“乔木进城”三年行动，打造更多城市“氧吧”，让“岳阳绿”成为我们城市的鲜明底色，让“岳阳蓝”成为我们城市的靓丽名片，让“岳阳人”可以惬意自由深呼吸！

六、坚持风险联防联控，筑牢岳阳发展的“防火墙”

风险防控是一项底线工作。我们要牢固树立忧患意识，切实增强工作的预见性、预警性，注重解决源头性、基础性问题，确保社会长治久安，确保人民安居乐业。

严格防控金融债务风险。狠抓金融生态整治，加快诚信体系建设，严厉打击恶意逃废金融债务行为，严肃处置民间非法集资行为，突出抓好公职人员参与非法集资专项整治，确保不发生系统性、区域性金融风险。狠抓政府债务风险防控，主动转变发展思路，正确处理好上项目、抓发展与防风险、化债务的关系，充分运用市场手段破解项目融资难题。严禁新上超出财政实际承受能力的“高大上”项目，严禁新上非必要性、非紧迫性、非公益性的“功利型”项目，坚决防止盲目举债建设，坚决杜绝大手大脚花钱，坚决严控政府债务率。

严格防控安全生产风险。按照“党政同责、一岗双责、失职追责”的要求，严格强化党政领导责任、属地管理责任、部门监管责任和企业主体责任。加强安全生产监管，实施安全生产有奖举报制度，组织开展安全生产隐患大排查大整治，突出抓好道路交通、危化物品、公共场所等重点领域安全监管，严格执行安全生产“八个断然措施”，坚决杜绝重特大安全事故发生。

严格防控社会稳定风险。发展是硬道理，稳定也是硬道理，我们要坚持发展和稳定“两手抓、两手硬”。提高社会治理能力，推行“12345+110+网格化”社会治理新模式，促进政府治理和社会调节、居民自治良性互动。加强社会治安防控，严厉打击暴力性、侵财性、团伙性和涉毒、涉黑、涉恶、涉赌等各类违法犯罪行为；组织开展信访“三无单位”创建活动，加大信访积案化解力度。通过织牢织密社会治理“保障网”，压紧压实社会安全“稳压器”，进一步提升岳阳城市的和谐指数、幸福指数。

七、坚持成果共建共享，提高岳阳发展的“普惠度”

改善民生是最大的政绩。我们要更加注重在发展中补齐民生短板、托住民生底线，让广大人民群众平等享有更多的发展成果。

强力推进脱贫攻坚。消除贫困既是我们的庄严承诺，更是我们的历史责任，离目标越近越要保持定力、加速冲刺。深入推进脱贫攻坚大会战，严格落实中央“五个一批”“六个精准”“七个强化”等工作要求。进一步抓好易地搬迁、产业扶贫、政策兜底、基础建设等“十大行动”，持续提高贫困地区、贫困群众自我发展的内生能力，加快攻克深度贫困堡垒；进一步严格扶贫资金监管，对扶贫资金核发、使用、管理进行全时段监管，确保每一分钱都用在“刀刃上”；进一步探索建立稳定脱贫的长效机制，既防“拖延病”，又防“急躁症”，既不降低标准，也不吊高胃口；进一步强化各级党政一把手、属地单位、村支“两委”、驻村扶贫队等扶贫责任，继续实行“三走访三签字”，做到逐户销号、脱贫到人、群众认账。确保2018年平江县顺利“摘帽”、109个贫困村整体退出、综合贫困发生率降到2%以下，坚决做到脱真贫、真脱贫。

全面繁荣社会事业。坚持教育优先，继续抓好中心城区中小学校“六建六扩”，加快推进中小学幼儿园规划建设，促进城乡义务教育一体化；深化职业院校产教融合、校企合作；推动学前教育、特殊教育、民办教育等有序发展。推进健康岳阳建设，加快整合市一医院与市三医院，抓好市妇女儿童医院、市中医院基本医疗大楼、市二医院全科医生培养大楼建设；深化医药卫生体制改革，完善分级诊疗制度，加快医联体建设。严格按照“四个最严”要求狠抓食品药品监管，确保“舌尖上的安全”。加快老龄事业和产业发展，扎实推进全国第二批居家和社区养老服务改革试点。广泛开展全民健身运动。深化国家公共文化服务体系示范区建设，积极实施“文化惠民工程”和“文化名家工程”，为岳阳人民提供更加丰富的“精神食粮”。

认真办好民生实事。实施积极的就业政策，新增城镇就业5.2万人、创业主体2.8万个。坚持住房的居住属性，加快建立多主体供应、多渠道保障、租购并举的住房制度，全面推进“五个5”惠民工程，稳定房地产市场信心和预期，坚决将市场房价控制在合理区间。积极构建中心城区入学、医疗、休闲、办证、警务“五个一公里”公共服务圈，让市民在“家门口”享受更多、更好、更便捷的公共服务。建成义务教育标准化教学点62个、农村公办幼儿园18所。加强基层综合文化服务中心建设，确保覆盖率达到100%。加大严重低洼渍水地段整治、农贸市场改造力度。加快公共停车场、停车泊位、人行天桥、地下人行通道建设。实施全民参保登记计划，稳步提高最低生活保障和特困供养标准，做好被征地农民社会保障工作。

完成全年目标任务，必须切实加强政府自身建设。我们将始终保持绝对忠诚的政治本色。大力弘扬平江起义革命精神，以举办平江起义90周年纪念活动为契机，进一步坚定理想信念和政治立场。着力强化“四个意识”，扎实开展“不忘初心、牢记使命”主题教育，进一步增强对习近平新时代中国特色社会主义思想的情感认同、理性认同和实践认同，一步一个脚印走好新时代岳阳发展的“长征路”。始终保持一心为民的公仆本色。大力弘扬忧乐精神，积极践行“以人民为中心”的发展理念，把人心向背作为最大的政治，多干顺民心、合民意的事，坚决不搞劳民伤财的“形象工程”；多干打基础、利长远的事，坚决不搞急功近利的“短期行为”；多干求实效、出实绩的事，坚决不搞哗众取宠的“虚假政绩”，以脚踏实地的工作作风和业绩，赢得广大群众的信任和拥护。始终保持实干担当的先锋本色。大力弘扬求索精神，继续苦干实干加油干，在推动岳阳发展上，坚持实事求是，不喊脱离实际的“口号”；坚持开拓创新，不走因循守旧的“老路”；坚持较真碰硬，不打推诿回避的“太极”；坚持尽职尽责，不当坐而论道的“看客”，说了的坚决做到，定了的坚决执行，真正让责任上肩，把担子担硬，将工作做实。始终保持公而忘私的奉献本色。大力弘扬骆驼精神，牢记“党员干部身份是公职公仆、职责是公务公干、维护的是公利公益”。紧紧围绕一个“公”字开展工作、推动发展，自觉做到公权为民、公正处事，切实做到奉公守法、秉公执法，积极做到克己奉公、夙夜在公，与全市人民一道协力同心、砥砺奋进，努力将岳阳的美好蓝图变成美丽现实。始终保持清正在心的清廉本色。严格执行中央“八项规定”及实施细则精神，坚定不移地反“四风”、转作风、正行风，严格落实党风廉政建设“一岗双责”，全面强化重点领域、关键环节和要害岗位监管，严格做到多“设防”、不“撤防”，知敬畏、守底线，以清廉形象凝聚起岳阳新增长极建设的磅礴力量。

各位代表，新时代开启新征程，新时代呼唤新作为。让我们紧密团结在以习近平同志为核心的党中央周围，在中共岳阳市委坚强领导下，以永不懈怠的精神状态、一往无前的奋斗姿态，为全面建成小康社会、加快建设湖南发展新增长极而努力奋斗！
